# Utilization of Sulfonated Waste Polystyrene-Based Cobalt Ferrite Magnetic Nanocomposites for Efficient Degradation of Calcon Dye

**DOI:** 10.3390/polym14142909

**Published:** 2022-07-17

**Authors:** Vennila Srinivasan, Vasam Sumalatha, Adhimoorthy Prasannan, Sankar Govindarajan

**Affiliations:** 1 Department of Polymer Chemistry, University of Madras, Guindy Campus, Chennai 600025, India; vennilasvr93@gmail.com; 2 Department of Physics, Indian Institute of Technology-Madras, Chennai 600036, India; lathavasam28@gmail.com; 3 Department of Materials Science and Engineering, National Taiwan University of Science and Technology, No. 43, Section 4, Keelung Road, Taipei 10607, Taiwan

**Keywords:** Calcon dye, cobalt ferrite, sulfonated waste polystyrene, polymeric magnetic nanoparticles, dye degradation

## Abstract

We presented a simple and efficient method for making a polymer–metal nanocomposite using various amounts of cobalt ferrite magnetic nanoparticles (CoFe_2_O_4_ MNp) with sulfonated waste polystyrene (SWPS) and utilized for Calcon dye degradation. The MNp was encapsulated with SWPS to avoid agglomeration and maintain its smaller size. ATR-FTIR, Raman spectroscopy, X-ray diffraction (XRD), thermogravimetric analysis (TGA), dynamic light scattering (DLS), field emission scanning electron microscopy (FESEM), high-resolution transmittance electron microscopy (HR-TEM), atomic force microscopy (AFM) and solid UV were used to analyze the produced polymeric magnetic nanoparticles (SWPS/MNp). As the MNp loading increases, the average particle size decreases. For Calcon dye degradation, SWPS/MNp (20 wt%) was utilized with a smaller average particle size, and the structural changes were detected using a UV-Vis spectrophotometer. As a result, the Calcon dye’s characteristic absorbance peak at 515 nm was red-shifted to 536 and 565 nm after 5 min, resulting in a color shift from dark brown to light blue that could be seen with the naked eye. A strong linear correlation was found between the red-shifted absorbance and the concentration of dye solution over the range of 10–100 ppm under optimal conditions. The proposed dye degradation process is simple, efficient, and environmentally friendly and has been successfully used to purify organic azo-dye-containing water.

## 1. Introduction

Water is the most essential substance to ensure survival on the planet for living creatures’ fundamental activities. Unfortunately, due to population increase, industrialization, civilization, domestic and agricultural activities, and other geological and environmental changes, the water quality of our aquifers continues to worsen. As a result, water pollution has become a significant issue in today’s world, affecting all living things, as well as domestic, recreational, fishing, transportation, and other commercial operations [1]. Chemical and environmental engineers are concerned about the significant source of water pollution caused by poisonous organic dyes and pigments due to the excessive release of pollutants into the surrounding environment due to increased industrialization and urbanization. More than 7 × 10^5^ tonnes of dyestuffs are manufactured yearly, with over 100,000 commercially accessible dyes released into water sources [2]. Even at low doses, most dyes are vivid and poisonous [3]. These colorful and poisonous dyes pollute both the surface and groundwater. Because of their synthetic origins and complex aromatic compounds, they are difficult to remove from wastewater. Most of them are not biodegradable in typical environmental settings and have mutagenic, carcinogenic, and allergenic effects on biota and humans [4].

Azo dyes, the most frequently used anionic azo dye in the textile, food, leather, paper, and pharmaceutical industries [5], are the most widely used synthetic colorants and have caused water, and soil-based environmental problems. Due to its high-water solubility and resistance to light, organic solvents, chemicals, and potent oxidizing agents, it is susceptible to microbial attack. Traditional wastewater treatment makes it difficult to break down and remove azo colors from water [6]. Although wastewater treatment chemicals such as alum, activated carbon, and ferric chloride (FeCl_3_) are available, they are costly and do not decrease dye toxicity [7]. This paper introduces magnetic polymer nanoparticles as a dye degradation alternative to address these issues. This method only uses visible light and oxygen in the air, producing reactive oxygen species [8]. This dye degradation process ends with nontoxic final products, an advantage of our new technology over conventional methods [9].

Several reports have been published on the removal, separation, and determination of dyes using various magnetic nanoparticles [10]. In particular, CoFe_2_O_4_ with a spinel structure exhibits interesting magnetic, magneto-resistive, and magneto-optical properties potentially used for a broad range of applications [11]. Even though they were designed to degrade dye from aqueous solutions, their usage in dye removal is limited due to several issues, including aggregation in the solution and poor surface accessibility, resulting in low degrading effectiveness [12]. This problem can be solved by surface modification of CoFe_2_O_4_ as an eco-friendly material with a low cost, a water-stable dispersing material, and a good degrading capacity. To prevent agglomeration between the MNp, sulfonated waste polystyrene (SWPS) was added during or after the preparation of CoFe_2_O_4_ MNp. Controlling the size and shape of nanoparticles allows this method to modify their physicochemical properties [13]. The SWPS is a protective polymer because it has the desired aqueous solution characteristics and contains positive -CH_2_ groups and sulfonated functional groups that can be absorbed and create a complex with metal ions [14]. The SWPS chain is thought to have adsorbed on the surface of the magnetic core of CoFe_2_O_4_ MNp, forming a shell. It has a highly selective dye degradation ability [15].

Several papers on the adsorption of organic azo dyes from aqueous solutions employing SWPS and different polymers coated with CoFe_2_O_4_ MNp are available [16,17,18,19,20]. However, to our knowledge, no study has been published on removing azo dye from an aqueous solution utilizing the SWPS/CoFe_2_O_4_ MNp degrading technique. Firstly, we use a co-precipitation approach to make CoFe_2_O_4_ MNp and concentrated sulfuric acid to make SWPS from waste polystyrene (WPS). The influence of SWPS on the structural, particle size, thermal, and morphological characteristics of MNp is reported using these synthesized materials and varied concentrations of CoFe_2_O_4_ MNp. Furthermore, the organic pollutant of Calcon dye was degraded using SWPS/MNp, and we also looked into the effects of contact time and initial dye concentration on the degradation mechanism.

## 2. Experimental Section

### 2.1. Materials

Waste polystyrene foam (WPS) was obtained from IFB microwave oven packaging material, which is purified by the re-precipitation method before being used. Ferric nitrate (Fe(NO_3_)_3_.9H_2_O) (LR grade, S.D FineChem Ltd., Chennai, India), cobaltous nitrate hexahydrate (Co(NO_3_)_2_.6H_2_O) (AR grade, S.D FineChem Ltd., Chennai, India), sulfuric acid (H_2_SO_4_; 95–97%, ISO grade, SRL, Chennai, India), dichloromethane (DCM; Extra pure, AR grade, SRL, Chennai, India), sodium hydroxide (NaOH; AR grade, SRL, Chennai, India), tetrahydrofuran (THF; Extra pure, AR grade, SRL, Chennai, India), concentrated hydrochloric acid (Con. HCl; ACS grade, Fisher Scientific, Chennai, India), ethanol (EtOH; 99.9%, AR grade, Analytical CSS reagent, Chennai, India) and Calcon dye (Loba Chemie Pvt Ltd., Chennai, India) were purchased and used.

### 2.2. Measurements

The structure of the synthesized material was confirmed by a FTIR and Raman spectrometer. FTIR analysis was performed using Nicolet iS50 FTIR (Thermo Nicolet, Chennai, India) equipped with attenuated total reflectance (ATR) mode within the spectral range of 4000–400 cm^−1^ with a resolution of 4 cm^−1^ and an average of 32 scans. Raman spectral analysis was performed using an XploRAPLUS Raman microscope (Horiba Scientific, Chennai, India) in the spectral range of 1000–100 cm^−1^ with 532 nm line of a He–Ne laser as the excitation source. A powder XRD study was carried out on a Bruker D2 PHASER (Taipei, Taiwan) diffractometer operating at 30 kV with a Cu Kα (λ = 0.15418 nm) target, and the patterns were recorded at room temperature in the angular range of 10–80° (2θ).

The average particle size and zeta potential of the polymeric MNp have performed in the range of 0.3 nm to 8.0 μm and −200 to +200 mV, respectively, using DLS SZ 100 Horiba Scientific (Horiba Scientific Instruments, India) equipped with He–Ne laser (λ = 633 nm) and operated at a scattering angle of 173°.

The roughness of the polymeric MNp was studied using AFM (ASTLUM Research, Chennai, India) analyzer. The synthesized polymeric MNp were dispersed entirely in EtOH by keeping sonication for 5–10 min and then coated on a glass plate and dried. The measurement was carried out at room temperature using non-contact mode with a scan size of 3μm × 3μm × 65 nm.

FESEM (JEOL JSM-6500F, Taipei, Taiwan) was used to study the surface morphology of polymeric MNp. The synthesized polymeric MNp were dispersed entirely in EtOH by keeping sonication for 5–10 min, then coated on silicon vapor and dried. Analysis was carried out in the range of 200 nm at a working distance of 10.8 mm, with a magnification of 15 kV. HR-TEM imaging was performed using FEI Tecnai G2 (Taipei, Taiwan) operating at 200 kV. The samples were prepared by sonication in EtOH, deposited onto a copper-coated carbon grid, and dried.

The thermal stability of polymer MNp was evaluated by a TGA analyzer (SDT Q600 TA Instrument, India). An amount of 5 mg of the sample was heated from 30 °C to 600 °C with a heating rate of 10 °C/min using ina N_2_ atmosphere.

Solid UV-visible spectroscopy (Jasco V-670 spectrophotometer, Taipei, Taiwan) was used to determine the band gap energy of bare MNp and polymeric MNp in the range of 200–1200 nm, and the liquid UV-visible spectrophotometer (Perkin Elmer, Lambda-650, Chennai, India) was used to measure the degradation of the Calcon dye.

### 2.3. Purification of Waste Polystyrene (WPS)

Styrofoam waste was chopped into small pieces (10 gm), dissolved in 50 mL of dry THF before being transferred to an addition funnel, and dropped into a 1L beaker with 500 mL of water. The precipitated Styrofoam was filtered and dried in a hot air circulating oven at 60 °C for 24 h, and this purified Styrofoam was used for further chemical modification.

### 2.4. Synthesis of Cobalt Ferrite Magnetic Nanoparticle (CoFe_2_O_4_ MNp)

The co-precipitation process createdcobalt ferrite magnetic nanoparticles (CoFe_2_O_4_ MNp). In a beaker, 10 mmol cobaltous nitrate hexahydrate (Co(NO_3_)_2_. 6H_2_O) was dissolved in 4 mL DI water having 1 mL concentration. HCl, meanwhile, 20 mmol ferric nitrate (Fe(NO_3_)_3_. 9H_2_O) was dissolved in 40 mL distilled water in a second beaker. Both solutions were heated separately to 50 °C with constant stirring. The two solutions were mixed and stirred at 50 °C for 30 min. This mixture of Co(NO_3_)_2_. 6H_2_O and Fe(NO_3_)_3_. 9H_2_O was added dropwise to a 200 mL 1M NaOH solution at 100 °C for 30 min while vigorously stirring. The solution was turned black and allowed to settle at room temperature at the end of the process. The residue was removed from the solution using a permanent magnet and carefully washed with distilled water and ethanol to eliminate any unreacted precursor nitrate or ions. After cleaning, the magnetic nanoparticle was dried in a vacuum desiccator at room temperature.

### 2.5. Synthesis of Sulfonated Waste Polystyrene (SWPS)

The precipitated polystyrene foam (5 g) dissolved in 50 mL of THF was taken in a 100 mL round bottom flask fitted with a magnetic stirrer. An equivalent amount of Con. H_2_SO_4_ (5 mL) was added slowly into the polymer solution under stirring at 20 °C. After addition, the reaction temperature was raised to 80 °C with continuous stirring for 3 h. After completion of the reaction, the polymer solution was poured slowly into excess of crushed ice under vigorous stirring. The precipitated polymer was filtered and washed several times with distilled water until pH was neutral, and then it was dried under a vacuum oven at 60 °C for 24 h.

### 2.6. Synthesis of SWPS-Based Polymeric Magnetic Nanoparticle (SWPS/MNp)

CoFe_2_O_4_ MNps were used to make SWPS/MNPs. An amount of 50 mL THF was used to dissolve 0.5 g of SWPS. The necessary quantity of MNp (10 wt% and 20 wt%) was suspended in 20 mL of THF and sonicated for 30 min to disperse. This suspended MNp was introduced to an SWPS solution dropwise for 3 h with mechanical stirring. Polymeric MNp was precipitated using hexane, subsequently filtered, dried at room temperature in vacuum desiccators, and ground into a fine powder (SWPS/MNp).

### 2.7. Preparation of Calcon Dye Stock Solution

Dissolved 50 mg of Calcon in 500 mL DI water to make the Calcon dye stock solution. The stock solution was then diluted with DI water to get five dye solutions with concentrations of 10, 30, 50, 70, and 100 ppm. These solutions were used to investigate the impact of initial dye concentration on the dye degradation process. For the 50 ppm concentrated dye solution, the time effect was also examined.

## 3. Result and Discussions

### 3.1. Synthesis of Polymeric Magnetic Nanoparticles (SWPS/MNp)

**Scheme 1:**
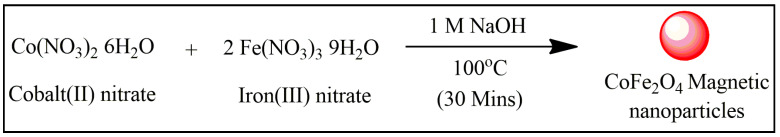
Formation of cobalt ferrite magnetic nanoparticle (CoFe_2_O_4_ MNp) using the co-precipitation method.

**Scheme 2:**
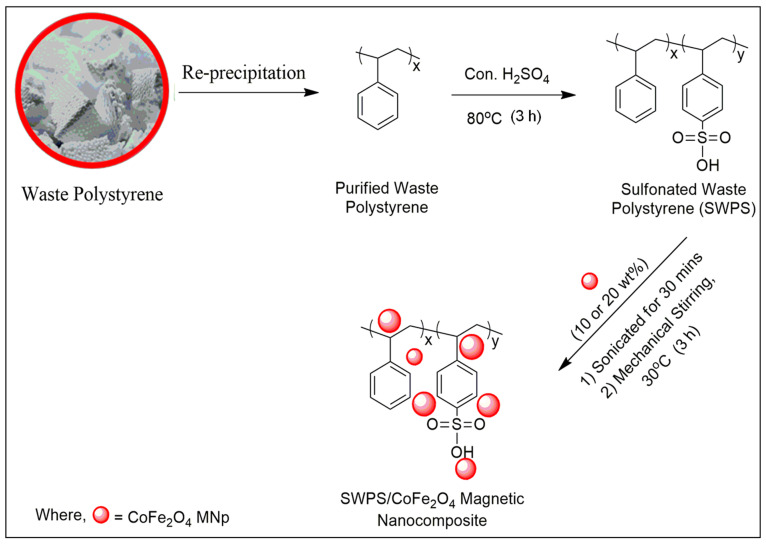
Synthesis of polymeric magnetic nanocomposites (SWPS/MNp) using different concentrated MNp (10 and 20 wt%).

Polymeric magnetic nanoparticles have recently attracted attention in the wastewater treatment domain, particularly for the removal of pesticides, halogenated compounds, and organic dyes from contaminated water, due to their numerous advantages, including high separation efficiency, ease of manipulation, favorable operating conditions, and easy specifically functional modification [21]. In this study, CoFe_2_O_4_ MNp was prepared by co-precipitation methods [22], and then partly SWPS was used as surfactants to prepare polymeric MNp because it is insoluble in water. The partly sulfonated polystyrene (SWPS) was prepared by reacting the waste polystyrene with concentrated sulfuric acid, and the degree of sulfonation was 61%, encouraging the reuse of hazardous polystyrene wastes [23]. The polymeric MNp (SWPS/MNp) was made with two different concentrations of concentrated CoFe_2_O_4_ MNp: 10 wt% and 20 wt%. Due to its comparatively small particle size, only 20 wt% polymeric MNp (SWPS/MNp) was used in the Calcon dye degradation process. Scheme 1 and Scheme 2 illustrate the formation of CoFe_2_O_4_ MNp and polymeric MNp.

### 3.2. Structural Evaluation of SWPS

**Figure 1:**
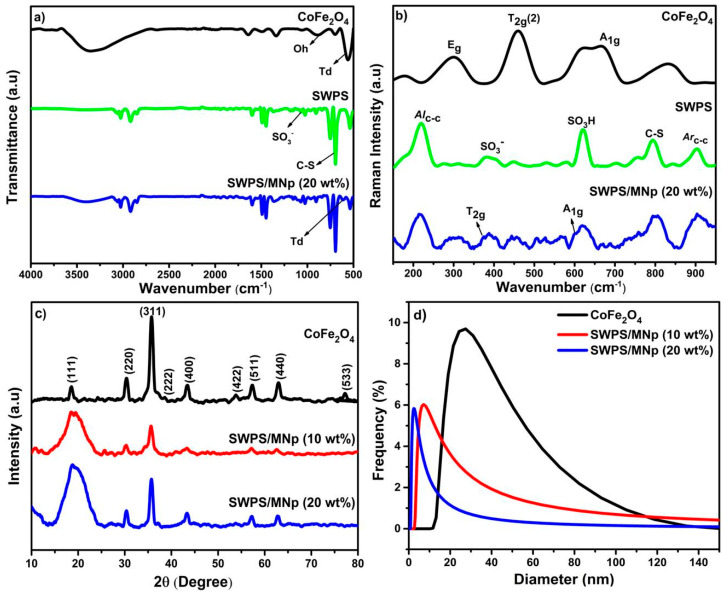
(**a**) ATR−FTIR spectrum; (**b**) Raman spectrum; (**c**) XRD patterns and (**d**) DLS measurements for size distributions.

Figure 1a showsthe ATR spectraof SWPS. The -OH group of sulfonic acid, which interacted with molecular water, is attributed to the broad absorption peak at 3436 cm^−1^. The strong absorption peaks that appeared at 1058, 696, and 536 cm^−1^ are attributed to the symmetric stretching of SO_3_^−^ groups, C-S stretching vibrations, and then the bonding of the sulfonic acid group in the para position, respectively. Previous researchers in SWPS [24] observed comparable results. The ATR spectrum of CoFe_2_O_4_ MNp is shown in Figure 1a. The Fe-O and Fe(Co)-O stretching vibrations at the octahedral (Oh) and tetrahedral (Td) sites of the inverse spinel structure are responsible for a prominent absorption peak at 887 and 564 cm^−1^ [25]. Because the surface bond force constant of the CoFe_2_O_4_ MNp increases after the MNp is incorporated into SWPS, the absorption peak at 576 cm^−1^ shifts to a higher wavenumber of around 610 cm^−1^ (Figure 1a).

Raman spectroscopy at room temperature in the wavenumber range of 100–1000 cm^−1^ was used to learn more about the structural characteristics of the polymeric MNp. In general, the spinal structured CoFe_2_O_4_ MNp with cubic Oh_7_ symmetry space group has 39 normal vibrational modes, of which five are active (e.g., 3T_2g_ + E_g_ + A_1g_) and twelve are inactive (e.g., T_1g_, 2A_2u_, 2E_u_, 4T_1u_ (IR), T_1g_ (acoustic), and 2T_2u_). Due to the random distribution of cations among A and B sites, defects-driven lattice distortions, and short-range ordering of cations on B sites, these extra Raman modes may be expected in inverse spinal structured ferrite MNp [26]. The metallic cations in the spinal structure are either surrounded by six oxygen ions, forming an octahedron, or by four oxygen ions, forming a tetrahedron. Figure 1b displays the Raman spectra for MNp, SWPS, and SWPS/MNp at ambient temperature. The prominent peaks at 177, 302, 458, 620, 664, 831, and 928 cm^−1^ in the Raman spectrum of MNpsare comparable with those described for other inverse spinal ferrite structures [27]. The asymmetric stretching of Fe(Co)-O bonds, related to the octahedral site mode that reflects local lattice effects in the octahedral sublattice, is attributed to the most intense T_2g_(2) Raman mode at 458 cm^−1^. The symmetry vibrations of the metal in the tetrahedral location of CoFe_2_O_4_ [28] are another powerful A_1g_ Raman mode at 664 cm^−1^ with a short shoulder peak at 620 cm^−1^. The vibrational modes of the C-C aliphatic chain, Sulfonate ion (SO_3_^−^), sulfonic acid (SO_3_H), para-substituted C-S stretching, and C-C aromatic ring, respectively, are attributed to the characteristic peaks at 225, 391, 627, 793, and 904 cm^−1^ in the Raman spectrum of SWPS. The Td (458 cm^−1^) and Oh site (664 cm^−1^) of M-O Raman modes shift towards a lower wavenumber after MNp is incorporated into SWPS [27]. The vibrational mode at 391 cm^−1^ in SWPS decreases as the MNp content increases, indicating the interaction between the sulfonic ion and metal cations in the B site.

XRD patterns of CoFe_2_O_4_ and polymeric MNp are shown in Figure 1c. The typical peaks of CoFe_2_O_4_ were observed at θ = 19°, 30°, 35°, 43°, 53°, 57°, 63°, and 77°, corresponding to the reflections from the planes (111), (220), (311), (400), (422), (511), (440) and (533) respectively, which are matched well with those from standard CoFe_2_O_4_ (JCPDS card no: 22-1086). Thus, the XRD patterns of the CoFe_2_O_4_ confirmed the single-phase cubic spinel structure, and the presence of solid and sharp peaks indicates a good crystalline nature. For SWPS/MNp, the XRD pattern shows that all the peaks belong to CoFe_2_O_4,_ and then the additional broad peak was observed at θ = 10° and 18°, which may be attributed to the overlap between SWPS and MNp. A similar result was reported by Morsi et al. [14] in SPS/CoFe_2_O_4_ nanocomposites prepared by the sol–gel method.

### 3.3. Particle Size and Morphological Studies

Dynamic light scattering (DLS) monitors the particle size, and the colloidal stability of MNp with spherical or anisotropic shapes is monitored using dynamic light scattering (DLS). DLS cannot distinguish between inorganic and organic materials and measures overall particle size [29]. The hydrodynamic size of the MNp, SWPS/MNp’s (0, 10, and 20 wt%) in distilled water (DW)is depicted in Figure 1d. The observed mean particle size of the MNp is 41.8 nm with a large particle distribution, and the mean particle size of SWPS/MNp reduces from 20.5 nm to 10 nm. The decrease in the average particle size of magnetic nanoparticles was attributed due to the stabilizing/capping ability of the sulfonated groups existing on the surface of SWPS. When distributed in DW, CoFe_2_O_4_ nanoparticles agglomerate and have a non-uniform size distribution, whereas SWPS/MNPs have a relatively uniform and narrow size distribution over MNp. According to zeta potential data, SWPS/MNp (20 wt%) is more stable in the colloidal system than other nanoparticles because their surface reduces inter-particle interactions and avoids agglomeration between them, as shown in Table 1.

**Figure 2:**
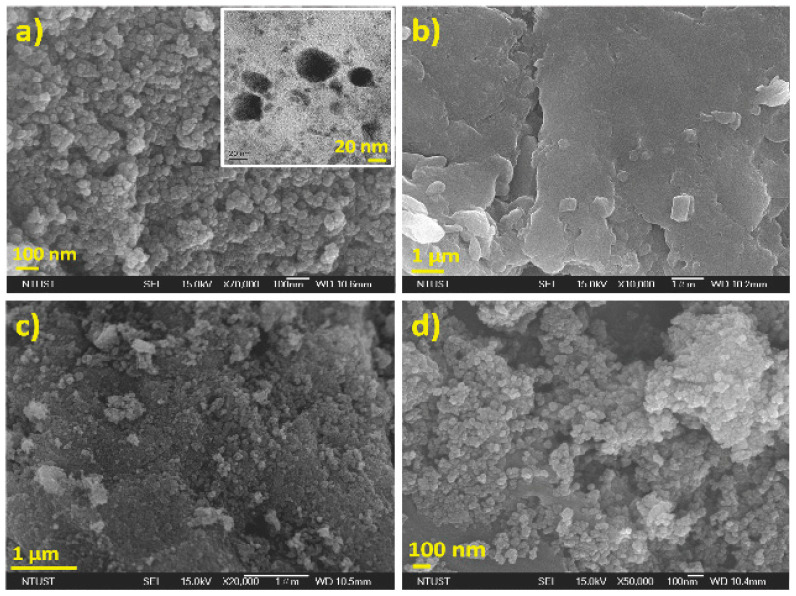
FE−SEM images of (**a**) CoFe_2_O_4_ (Inset shows TEM image), (**b**) SWPS, (**c**) SWPS/MNp (10 wt%) and (**d**) SWPS/MNp (20 wt%).

**Figure 3:**
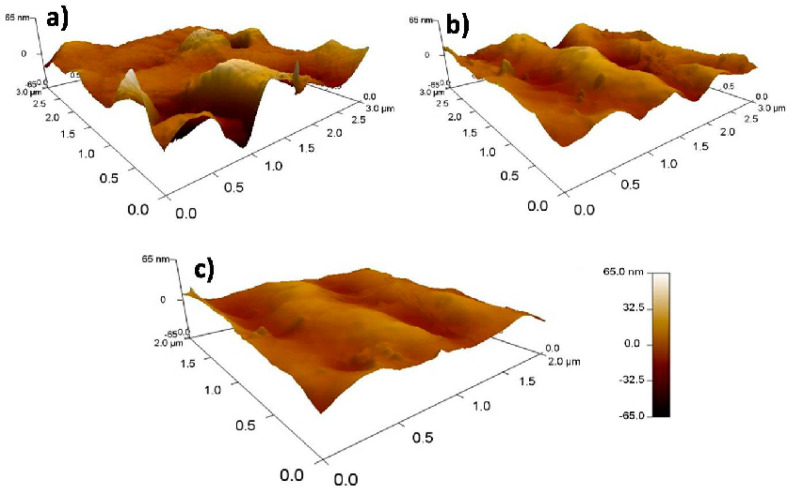
AFM images of (**a**) SWPS, (**b**) SWPS/MNp (10 wt%) and (**c**) SWPS/MNp (20 wt%).

Figure 2a–d show the surface morphology of CoFe_2_O_4_, SWPS, and SWPS/MNp (10 and 20 wt%) using FE-SEM. SWPS has a flat shape in SEM images; however, when MNp is added to SWPS, the shape changes to spherical due to surface ionic interactions between SWPS and MNp. HR-TEM images of CoFe_2_O_4_ in Figure 2a (inset) reveal that the nanoparticles were in the size range of less than 20 nm, which are closely packed spherical particles with irregular boundaries. From Figure 2a, the surface morphology of CoFe_2_O_4_ is clearly seen as irregular, and some of the particles are agglomerated, which may be attributed to the magnetic dipole interaction between the nanoparticles and high surface energy. The addition of SWPS showed the distribution of nearly uniform size CoFe_2_O_4_ nanoparticles. The particle size is significantly reduced in the following order: SWPS/MNp (20 wt%) < SWPS/MNp (10 wt%) < CoFe_2_O_4_, which indicates the reduction in the interaction between magnetic nanoparticles since the polymer added during the synthesis process reduces the inter-particles interaction and therefore prevents the agglomeration between them [14,17]. The surface roughness of the SWPS and SWPS/MNp was measured using atomic force microscopy (AFM), as illustrated in Figure 3a–c. The SWPS has a surface roughness of 30.38 nm, while the SWPS/MNp has a roughness of roughly 24.11 nm. Interparticle interactions occur on the surface of SWPS, as evidenced by the decreased surface roughness.

### 3.4. Thermal Stability

**Figure 4:**
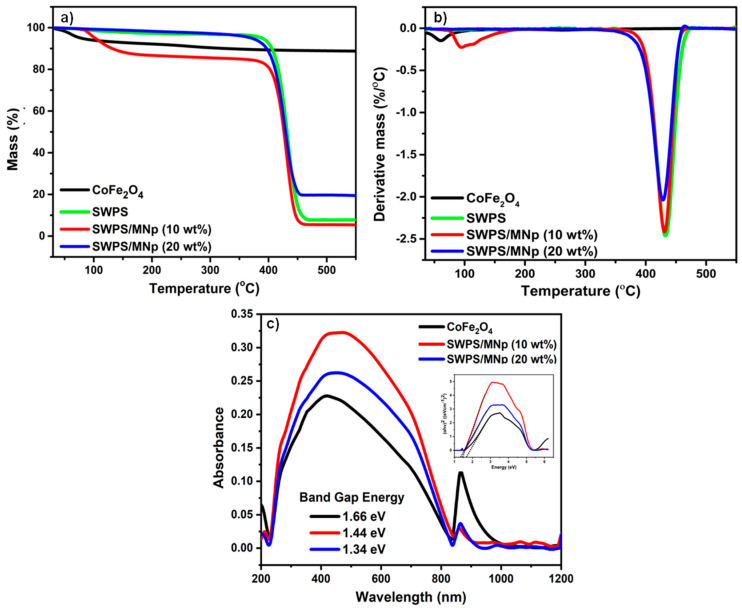
(**a**) TGA curves; (**b**) DTG curves and (**c**) solid UV spectrum (inset shows Tauc plot).

Thermogravimetric analysis of CoFe2O4, SWPS, and SWPS/MNPs were performed in the temperature range of 30–550 °C under an N_2_ atmosphere, and changes in polymeric substances mass loss were recorded. Figure 4a,b shows the TGA and DTG curves of MNp, SWPS, and SWPS/MNp. SWPS shows only one weight loss (92%) due to the decomposition of sulfonated Styrofoam, even though the initial weight loss starts around 200 °C, the significant weight loss starts above 380 °C. Effective weight loss of virgin sulfonated polystyrene typically occurs at about 330 °C. However, in this case, waste Styrofoam the significant weight loss starts above 380 °C. This may be due to small quantities of unremoved additives used to prepare the Styrofoam [30,31]. The gravimetrical analysis of the CoFe_2_O_4_ MNp revealed two different weight losses, the first at low-temperature range due to adsorbed water molecules and the other at high temperature in the range of 200–550 °C giving rise to significant weight loss around 324 °C, affirming the phase purity of the CoFe_2_O_4_. Significantly increased weight loss (77.3%) was observed with the onset temperature of 384 °C for SWPS/MNp. It can be deduced that incorporating MNp onto the SWPS enhances the surface-to-volume ratio of MNp while lowering the weight loss percentage, implying that the particle size of the SWPS/MNp should be smaller than that of CoFe_2_O_4_ MNp.

### 3.5. Determination of Band Gap Energy

Figure 4c represents the UV-visible spectrum of synthesized magnetic nanoparticles in the range of 200–1200 nm. The results were analyzed using the Tauc plot model to identify the band gap values of the samples. The calculated band gap energy values were varied from 1.66 eV, 1.44 eV and 1.34 eV for CoFe_2_O_4_, SWPS/MNp (10 wt%) and SWPS/MNp (20 wt%). Generally, the band gap energy value is directly proportional to the particle size, which is a highly efficient catalyst for dye degradation [32]. As a result, SWPS/MNp (20 wt%) was used as a catalyst for dye degradation processes.

### 3.6. Degradation of Calcon Dye

**Scheme 3:**
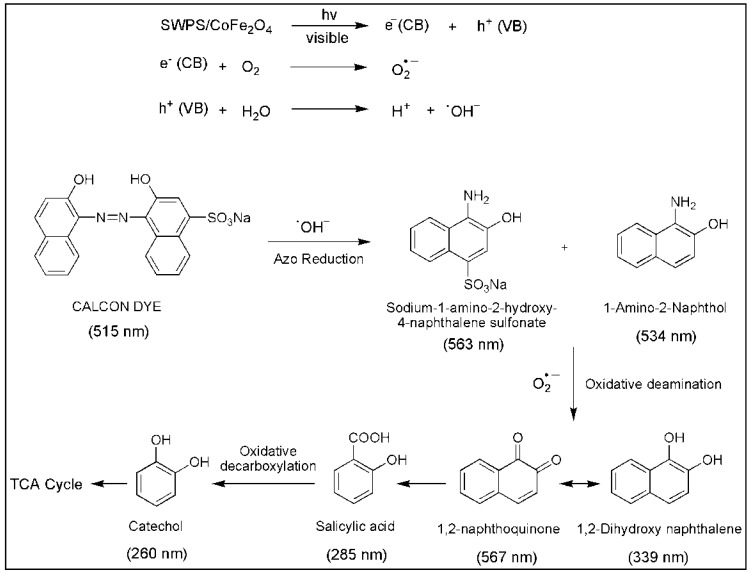
Mechanism of Calcon dye degradation using SWPS/MNp.

SWPS/MNp (20 wt%) has been used to degrade hazardous anionic azo dye, such as Calcon, as a test dye. The degradation process was carried out in a 100 mL RB flask, with 50 mg of SWPS/MNp added to 50 mL of Calcon dye aqueous solution (50 ppm) and kept at room temperature with constant stirring. The mechanism of azo dye degradation over SWPS/MNp is as follows [33]. The first step was to adsorb dye onto the surface of SWPS/MNp and generate electron-hole (e^−^-h^+^) pairs in SWPS/MNp; the corresponding process was clearly shownin Scheme 3. The electrons generated in the SWPS/MNp conduction band (CB) interact with the oxygen molecules adsorbed on SWPS/MNp to form superoxide anion radicals (O_2_^−^), and the holes generated in the valence band of MNPs reacted with the −OH groups existing in the surface of the dye molecule and produces hydroxyl radicals which are highly reactive in nature. The holes (photo-generated) are involved in the dissociation of water molecules in the reaction to produce free radicals. The existing hydroxyl radicals (OH) and superoxide radicals (O_2_^−^) in the reaction solution would directly interact with the azo group of the Calcon dye, which is adsorbed on the surface of SWPS/MNp and thus, the degradation mechanism progresses to produce different degradation products.

Initially, when the photocatalytic degradation process began, the characteristic absorbance peak of Calcon dye at 515 nm was red-shifted to 536 and 565 nm, indicating that 1-amino-2-naphthol and sodium 1-amino-2-hydroxy-4-naphthalene sulfonate was formed via azo reduction [34,35]. In brief, the photocatalytic degradation process mainly involves three organic processes viz., azo reduction, oxidative deamination, and oxidative decarboxylation. The term photocatalytic degradation was mentioned as photons are more significant in generating the electron-hole (e^−^-h^+^) pairs in the SWPS–MNp nanocomposite. The Calcon dye was converted to different organic products such as Sodium-1-amino-2-hydroxy-4-naphthalene sulfonate (563 nm), 1-amino-2-naphthol (534 nm), 1,2-naphthoquinone (567 nm) and its hydroxyl counter-part 1,2-dihydroxy naphthalene (keto-enol tautomeric product) (339 nm), salicylic acid (285 nm) and catechol (260 nm) and the progress of conversions were noticed from the changes in the wavelength of UV-Visible spectra [35,36,37]. As a result, the color of the Calcon dye solution changed from dark brown to light blue, which could also be seen with the naked eye. A similar mechanism was reported in the biodegradation process [35]. Table 2 lists the other catalysts used in the literature to degrade Calcon dye. The results are not directly comparable because of different experimental conditions such as pH, temperature, and time but can be indirectly evaluated based on their degradation performance. The SWPS/MNp (20 wt%) reported in this work is a simple, low-cost, and more effective heterogeneous catalyst for dye degradation, which does not require heating or maintaining particular pH compared to the other reported metal catalysts.

### 3.7. Effect of Contact Time

**Figure 5:**
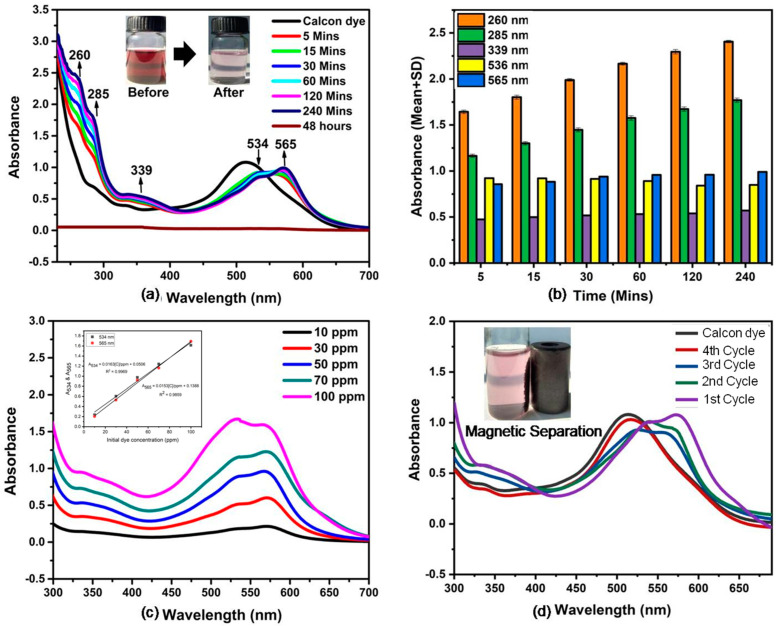
(**a**) UV−vis spectrum of Calcon dye degradation with varies time; (**b**) comparative plot of degraded products using contact time; (**c**) Effect of initial dye concentration and (**d**) UV spectrum of recycled SWPS/MNp (20 wt%) with magnetic separation.

In this investigation, 50 mg of SWPS/MNp were dispersed in 50 mL of 50 ppm aqueous dye solution with constant stirring for a range of 5–240 min at room temperature, and the UV spectra were depicted in Figure 5a. The UV absorbance peak at 536 nm decreases over time, indicating that the generated 1-amino-2-naphthol undergoes oxidative deamination to yield 1, 2-naphthoquinol, which is detected by UV-vis analysis with an increase in absorbance peak strength at 565 nm. Figure 5b depicts a comparative plot illustrating the effect of contact time on Calcon dye degradation with error bars. The absorbance peak at 536 nm did not change after 60 min, while the other absorbance peaks (260, 285, 339, and 565 nm) had significantly increased. The constant peak at 534 nm indicates that the azo reduction is complete, whereas the other growing peaks relate to the continued oxidation of the reduced product during the degradation process. The extra three absorbance peaks (260, 285, and 339 nm) increase with increasing contact time, whereas the absorbance peak at 534 nm decreases, implying efficacy in Calcon dye degradation. The absorbance peaks vanish with time, resulting in a colorless liquid, indicating that the molecules have entirely degraded.

### 3.8. Effect of Initial Dye Concentration

The effect of Calcon dye concentration was explored by repeating trials for 60 min with varying initial Calcon dye concentrations (10, 30, 50, 70, and 100 ppm), and the resulting UV spectrum was presented in Figure 5c. An excellent linear relationship (correlation value R = 0.9969) was observed between the absorbance at 536 and 565 nm and the concentration of dye solution over the range of 10–100 ppm under optimal conditions. As the azo reduction and oxidative reaction in the degradation process progress, the initial dye concentration rises. However, in the case of a highly concentrated dye solution (100 ppm), the absorbance peak at 565 nm was lower than the absorbance peak at 536 nm because it has a large number of vacant surface sites in the early stages of azo reduction and the remaining vacant surface sites are challenging to occupy after some time due to the repulsive force between the Calcon dye adsorbed on the surface of SWPS/MNp and the solution phase. The initial dye concentration influences the degradation process.

### 3.9. Magnetic Separation and Reusable

One of the venerable benefits of using SWPS/MNp as a heterogeneous catalyst is the easy magnetic separation which ensures catalyst stability over multiple catalytic runs. For the recyclability experiment, the catalyst was separated using a magnet after completing the degradation reaction, which was reused four times. The UV spectra are shown in Figure 5d. A slight decrease in the catalyst activity was observed in the second and third cycles, which illustrates that SWPS/MNp is a magnetically separable heterogeneous catalyst that can be reusable.

## 4. Conclusions

To summarize, we effectively synthesized and characterized spherical-shaped CoFe_2_O_4_ MNp and SWPS/MNp with 10 and 20% MNp loading, the average particle size of 27.5–1.1 nm, and evaluated their performance in the degradation of azo dye. The degradation of the Calcon dye from an aqueous solution was caused by the smaller particle size of the SWPS/MNp, which is due to SWPS encapsulating MNp. The higher dye degradation efficiency of SWPS/MNp (20 wt%) supports the hypothesis that smaller particles lead to better dye degradation. It has also been discovered that as the contact time and initial dye concentration increase, the dye degradation efficiency improves, as determined by UV-vis spectroscopy. The method we proposed in this paper is a high standard method with several advantages, including the use of waste polystyrene, which reduces environmental pollution, avoids harmful gases, which are environmentally beneficial, and is a convenient and straightforward procedure that saves energy resources and costs in nanoparticle synthesis.

## Figures and Tables

**Table 1:** Size distribution and zeta potential from DLS measurements of CoFe_2_O_4_ MNpand SWPS/MNp (10 and 20 wt%).

S.No	Sample Code	Z-Average Size (nm)	Zeta Potential (mV)
1	CoFe_2_O_4_ MNp	27.5	−31.6
2	SWPS/MNp (10 wt%)	2.3	−43.7
3	SWPS/MNp (20 wt%)	1.1	−46.2

**Table 2:** List of metal catalysts in the dye degradation process.

S.No	Metal Catalyst (mg)	Experimental Conditions	Reference
1	Na-MnO_2_/SnO_2_(50)	Dark Temperature—Room; Time—24 h; pH: 3	[38]
2	Chitosan/Fe_3_O_4_ (50)	Temperature—25 °C; Time—240 min; pH: 3	[6]
3	ZnO (50)	UV irradiation (250 W); Time—260 min; pH: 4.5	[39]
4	SWPS/CoFe_2_O_4_ (50)	Natural Light; Temperature—Ambient; Time—240 min; pH: Natural	This work

## Data Availability

Not applicable.
